# Hormonal and nutritional regulation of alternative CD36 transcripts in rat liver – a role for growth hormone in alternative exon usage

**DOI:** 10.1186/1471-2199-8-60

**Published:** 2007-07-17

**Authors:** Louisa Cheung, Malin Andersen, Carolina Gustavsson, Jacob Odeberg, Leandro Fernández-Pérez, Gunnar Norstedt, Petra Tollet-Egnell

**Affiliations:** 1 Department of Molecular Medicine, Karolinska Institutet, Karolinska Hospital, 171 76 Stockholm, Sweden; 2 Department of Medicine, Atherosclerosis Research Unit, King Gustaf V Research Institute, Karolinska Institutet, Karolinska Hospital, 171 76 Stockholm, and Department of Biotechnology, AlbaNova University Center, Royal Institute of Technology, 106 91 Stockholm, Sweden; 3 Department of Clinical Sciences, Molecular and Translational Endocrinology Group, University of Las Palmas of G.C., Las Palmas of G.C., Canary Islands, Spain

## Abstract

**Background:**

CD36 is a multiligand receptor involved in various metabolic pathways, including cellular uptake of long-chain fatty acids. Defect function or expression of CD36 can result in dyslipidemia or insulin resistance. We have previously shown that CD36 expression is female-predominant in rat liver. In the present study, hormonal and nutritional regulation of hepatic CD36 expression was examined in male and female rats. Since alternative transcription start sites have been described in murine and human *Cd36*, we investigated whether alternative CD36 transcripts are differentially regulated in rat liver during these conditions.

**Results:**

Sequence information of the rat *Cd36 *5'-UTR was extended, showing that the gene structure of *Cd36 *in rat is similar to that previously described in mouse with at least two alternative first exons. The rat *Cd36 *exon 1a promoter was sequenced and found to be highly similar to murine and human *Cd36*. We show that alternative first exon usage is involved in the female-predominant expression of CD36 in rat liver and during certain hormonal states that induce CD36 mRNA abundance. Estrogen treatment or continuous infusion of growth hormone (GH) in male rats induced CD36 expression preferentially through the exon 1a promoter. Old age was associated with increased CD36 expression in male rats, albeit without any preferential first exon usage. Intermittent GH treatment in old male rats reversed this effect. Mild starvation (12 hours without food) reduced CD36 expression in female liver, whereas its expression was increased in skeletal muscle.

**Conclusion:**

The results obtained in this study confirm and extend our previous observation that GH is an important regulator of hepatic CD36, and depending on the mode of treatment (continuous or intermittent) the gene might be either induced or repressed. We suggest that the effects of continuous GH secretion in females (which is stimulatory) and intermittent GH secretion in males (which is inhibitory) explains the sex-different expression of this gene. Furthermore, a female-specific repression of hepatic CD36 in response to food deprivation was found, which was in contrast to a stimulatory effect in skeletal muscle. This demonstrates a tissue-specific regulation of *Cd36*.

## Background

Fatty acid translocase (FAT or CD36) is a cell-surface glycoprotein that functions as a multiligand receptor/transporter involved in various diverse physiological processes and disorders, including atherosclerosis, dyslipidemia, insulin resistance and diabetes (reviewed in [1]). In peripheral tissues active in fatty acid metabolism, such as muscle and adipose tissues, CD36 facilitates the uptake of long-chain fatty acids (LCFA) across the plasma membrane. A null mutation of *Cd36 *reduces fatty acid (FA) uptake rates and metabolism in these tissues [2], while its over-expression results in the opposing effects [3,4]. Although CD36-deficient mice display reduced triacylglycerol (TG) in muscle, lipid accumulation is increased in the liver. The latter is probably due to increased plasma FA concentrations and CD36-independent hepatic FA uptake. As a consequence of these effects, insulin sensitivity is enhanced peripherally, while it appears impaired in the liver [5]. Since muscle and adipose tissues shift to high glucose utilization in CD36-deficiency, hypoglycemia and hypoinsulinemia develop in the fasted state in CD36-deficient mice [6]. Also, over-expression of CD36 in muscle tissues is associated with hyperglycemia and hyperinsulinemia, as a result of enhanced FA utilisation and glucose sparing. Taken together, expression-levels of CD36 affect lipid and glucose utilization and insulin sensitivity in different tissues. Abnormal regulation of *Cd36 *expression might therefore contribute to the onset or severity of several metabolic diseases.

CD36 abundance is regulated at different levels, including gene expression, mRNA stability and protein expression in a cell- and tissue-specific manner. Different physiological conditions, where the nutritional and/or hormonal status of the individual is affected, have been shown to impact on CD36 levels in the plasma membrane. Regulation at the level of mRNA expression in skeletal muscle has been reported to include starvation, refeeding [7] and exercise [8], but only a few studies report the molecular mechanisms behind these effects. Although PPARα, PPARγ [9,10], PXR [11], NR4A (Nu77) [12] and Foxa2 (HNF3β) [13] have been shown to affect CD36 expression, no direct interaction of these transcription factors in the *Cd36 *promoter have been described. Furthermore, recent analyses of the *Cd36 *gene has revealed a complicated promoter structure with alternative transcription start sites [14,15]. Alternative promoter usage has been shown to contribute to tissue-specific regulation of CD36 expression in both mice and humans [14,15]. Although the alternative promoters are mapped in the human and murine *Cd36 *genes, less information is available for the rat gene.

Hepatic CD36 has not been considered physiologically important, due to low level of expression in the liver and the fact that hepatic lipid uptake is independent of CD36. Perhaps as a consequence of this, little is known about hepatic *Cd36 *gene regulation. However, we have recently shown that CD36 expression is female-predominant in rat liver [16]. It was also shown that the female pattern of growth hormone (GH) secretion induces CD36 mRNA levels in male liver, suggesting that there might be conditions where increased or decreased activities of hepatic CD36 is needed and that these conditions might be slightly different in male and female livers. The aim with the present study was to extend our knowledge about regulatory mechanisms behind hepatic CD36 expression. Since alternative CD36 transcripts have been described in mouse and human tissues, we investigated whether alternative transcription start sites might be involved in differentially regulating CD36 mRNA abundance in rat liver in different hormonal and nutritional states.

## Results and discussion

### Comparison between mouse and rat Cd36 gene structures

The mouse *Cd36 *gene has at least three characterized untranslated first exons and corresponding promoters, resulting in three major alternative transcripts [17]. Similarly, the human *Cd36 *gene can be expressed from five alternative first exons [14]. When the first exon of rat *Cd36 *([GenBank:NM_0031561]) is mapped to the genome browser it is equivalent to exon 2 in the human and mouse genes. Gene structures and exon distances are otherwise similar between mouse and rat. Since the rat genomic sequence is incomplete upstream of exon 2 (when extrapolated to the mouse and human gene structures), we looked for rat exon 1a and 1b in the UCSC genome database. A few ESTs showed high sequence homology to corresponding mouse exon 1b. However, the estimated location of rat exon 1a fell within regions where sequence information is unavailable.

**Figure 1:**
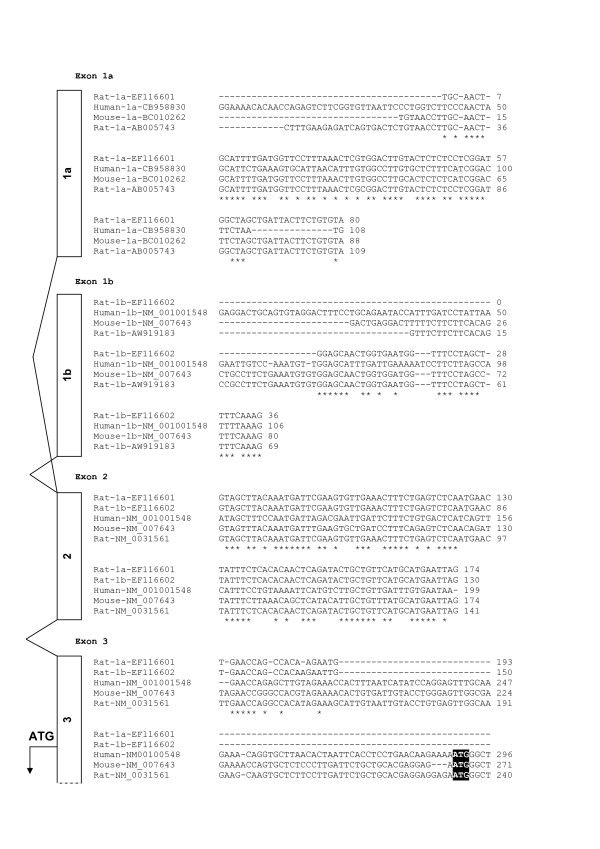
**The sequence of *Cd36***. Obtained sequences ([GenBank:EF116601] and [GenBank:EF116602]) were aligned to known sequences of *Cd36 *exon 1a, 1b, 2 and 3 using the multiple alignment program ClustalW version 1.83, available on the EBI website. The reverse strand is shown in mouse sequences. Base numbers specific to the individual sequences are presented. The transcription start site in exon 3 is bold and highlighted. Asterisks indicate bases which are identical among all three species.

To determine whether the rat *Cd36 *5'-untranslated region (UTR) is similar to that of the mouse, primers were designed from the common sequences between mouse and rat; for exon 1a according to [GenBank:BC010262] (mouse) and [GenBank:AB005743] (rat), and for exon 1b according to [GenBank:NM_007643] (mouse) and [GenBank:AW919183] (rat). PCR products derived from male or female rat hepatic cDNA, corresponding to regions from either exon 1a to exon 3 (1a-3) or from exon 1b to exon 3 (1b-3), were generated and sequenced. The amplified products appeared as single bands, as examined by gel electrophoresis, with the corresponding estimated sizes of 250 bp for 1a-3 and 150 bp for 1b-3. Upon sequencing, it was apparent that these products had different first exon sequences but identical exon 2 and 3 sequences ([GenBank:EF116601] and [GenBank:EF116602]). This finding suggests that the use of exon 1a and exon 1b is mutually exclusive in rat liver (Figure 1). Furthermore, high similarity scores between mouse and rat exon 1a-3 (92%) and 1b-3 (94%) sequences, suggest that the sequenced PCR products originating from rat liver are homologous to the corresponding mouse CD36 transcripts. Aligning human and rodent exon 1a and 1b sequences revealed low similarities.

**Figure 2:**
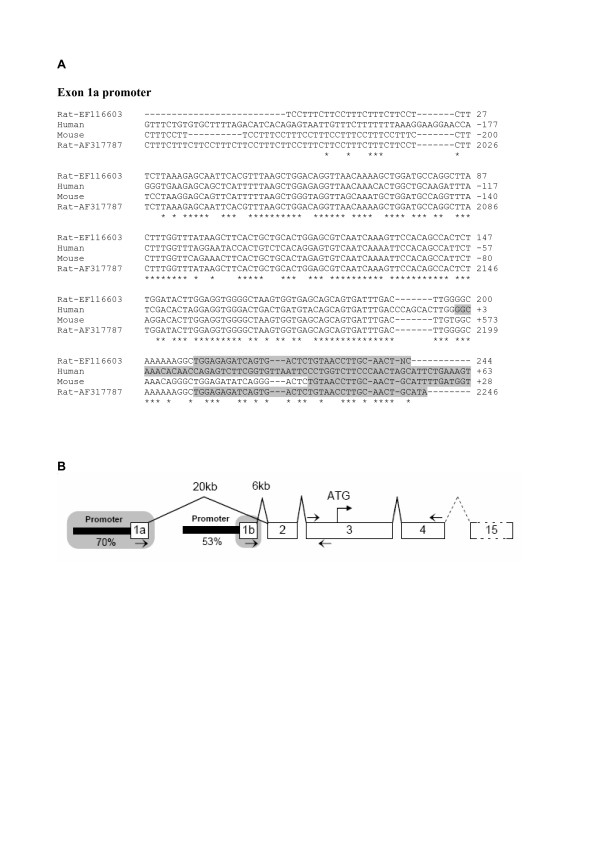
**The sequence of the *Cd36 *promoter**. A. Promoter sequences were extracted from UCSC genome browser upstream of *Cd36 *exon 1a from mouse [GenBank:BC010262] and human [GenBank:CB958830] and aligned to the previously described rat promoter [GenBank:AF317787] and the sequence obtained in this study [GenBank:EF116603]. Base numbers for [GenBank:AF317787] and [GenBank:EF116603] are specific to the respective sequences. For others, transcription start site of exon 1a (+1) is referred to as the first base of the sequence. The reverse strand is shown in mouse sequences. Corresponding exon sequences for the different species are shadowed. In rat, the exon 1a region was determined by aligning the promoter sequence with [GenBank:AB005743], where the first three bases were excluded due to inconsistency. B. Summary of murine *Cd36 *gene structure which consists of 15 exons. Exons 1a, 1b and 2, first part of exon 3, and exon 15 are untranslated regions (UTR). The sequences of exon 1a and its promoter as well as exon 1b were identified in this study (shaded area). The use of these two alternative first exons is mutually exclusive. The degree of sequence conservation among human, mouse and rat in the promoters (200 bp upstream) of exon 1a and exon 1b are presented. Approximate distances between exon 2 and the alternative first exons in mouse are also indicated. Arrows represent the position of exon-specific primers for real-time PCR analysis.

Since sequence information for rat *Cd36 *exon 1a and its promoter is limited, this promoter was cloned from rat liver DNA. Primers were designed corresponding to highly conserved genomic regions within the human and mouse exon 1a promoter. The amplified product was estimated to be 450 bp long with a sequence similar to that of the mouse 1a promoter region, as described by Sato *et al*. [17]. This sequence [GenBank:EF116603] was also identical to part of a cloned rat *Cd36 *promoter [GenBank:AF317787], not mapped in genome databases (Figure 2A). Alignment of these rodent promoter sequences and the human exon 1a promoter revealed 70% similarity in the region of -200 bp, whereas the corresponding promoter regions of exon 1b were slightly less similar (53%) (Figure 2B). This suggests that the regulatory function of these promoters might be evolutionarily conserved.

Tissue-specific expression patterns of alternative first exons of *Cd36 *have been described in mouse [15] and human [14], suggesting that the alternative first exons of the gene are regulated individually. According to Sato *et al.*, exon 1a is more expressed in liver than muscle, whereas the opposite is shown for exon 1b [15]. Comparing expression levels in human tissues active in FA metabolism, exon 1a is highest expressed in adipose tissue, followed by heart, skeletal muscle and liver, with a ratio of approximately 100 between adipose tissue and liver [14]. Exon 1b showed the same relative expression levels, but only 10 fold differences between fat and liver [14]. Whether males and females have different tissue-specific patterns of alternative first exons was not investigated in the studies referred to.

### Sex different expression of CD36 transcripts in mouse and rat liver

**Figure 3:**
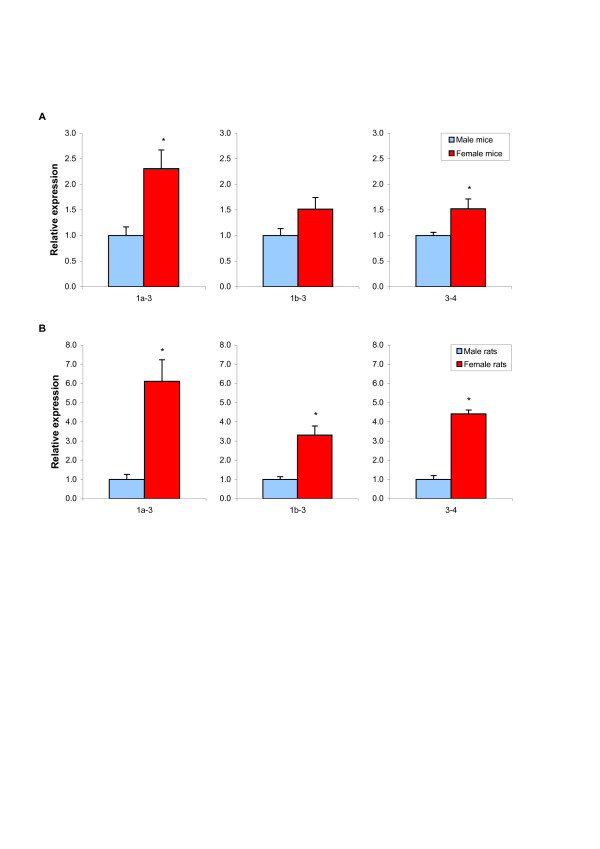
**Effects of sex on hepatic CD36 mRNA expression in mice and rats**. mRNA expression of CD36 was quantified by real-time PCR in livers from male or female mice (A) and rats (B). Exon-specific primers were used to measure the mRNA abundance of specific regions of the transcript, which were normalized to the mRNA level of GAPDH in mouse and that of RPLP0 in rat samples. Data are presented in relation to the expression level of the same transcript in male counterparts. The obtained data were analyzed using Student's T-test and bars represent the mean ± S.E. Asterisks indicate significant sex differences (p < 0.05).

We next wanted to determine a possible role for alternative promoter usage in the previously described sex-different expression of CD36 in rat liver. Expression levels of exon 1a and 1b were thus analyzed in male and female livers from rats and mice by real-time PCR and compared to exon 3–4, representing the protein coding region. As demonstrated in Figure 3, the exon 1a-3 transcript was significantly higher expressed in females as compared to males, in both species. Although similar results were obtained for exon 1b-3 and the coding region (exon 3–4), the sex-difference was most pronounced for exon 1a-3. A species difference in the degree of sex-dependence was observed for all transcripts measured, with larger differences in rats. Compared to males, female rats had 4.4 times higher expression of the exon 3–4 transcript in liver, whereas the difference was only 1.5 times in mice. Similarly, female rats had 6-fold higher levels of exon 1a-3 compared to males. In the mouse, female expression was only 2.3-fold higher compared to males.

### Hormonal regulation of hepatic CD36 expression

Many hepatic sex-differences in gene expression can be explained by sex-different levels of different hormones. Male rats exposed to estrogen display a "feminized" pattern of hepatic gene expression [18]. GH also exerts feminizing effects, but only if delivered in a continuous fashion that mimics the endogenous female secretory pattern of GH (GHc). A male pattern of hepatic gene expression can instead be induced by intermittent administration of GH (GHi) [19]. This can be explained by the fact that plasma GH profiles are sexually dimorphic in rodents. Male rats secrete GH in an episodic rhythm in which interpulse periods contain no detectable levels of the hormone. GH secretion in the female rat is also pulsatile, but can be characterized as "continuous" since hormone levels are always present in the circulation. Old age, which is associated with reduced pulse amplitude but unchanged trough GH values in male rats, has also been shown to "feminize" hepatic gene expression [20,21]. In mice, although the pulse frequencies are greater in females, GH levels in peaks and interpeaks are the same between males and females [22]. This might explain why some sex-dependent and GH regulated genes (such as CD36) are more sex-differentiated in rats as compared to mice.

**Figure 4:**
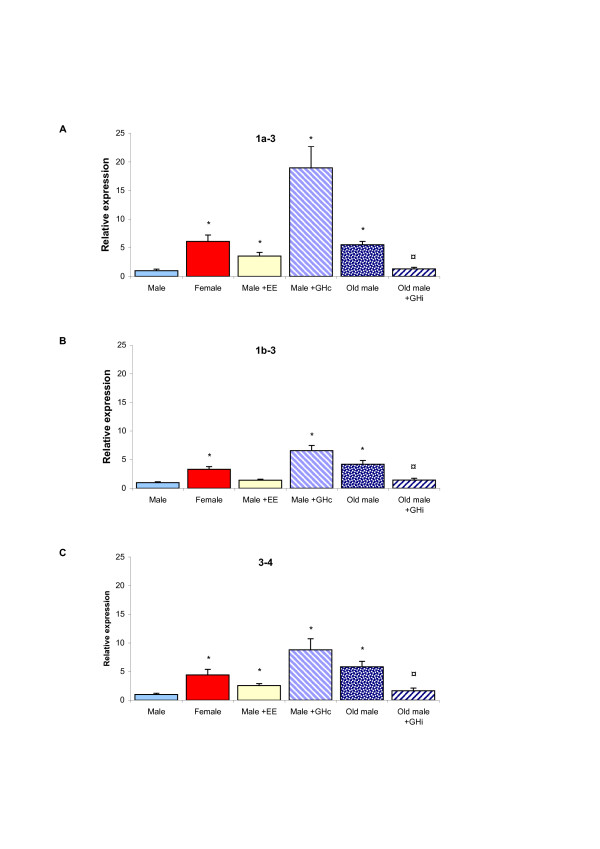
**Effects of different hormonal treatments on hepatic CD36 mRNA expression in male rats**. mRNA expression of CD36 exon 1a-3 (A), exon 1b-3 (B) and exon 3–4 (C) was quantified by real-time PCR in livers from males, females, males treated with continuous infusion of GH (GHc), males treated with injections of estradiol, old males or old males treated with twice daily injections of GH (GHi). Exon-specific primers were used to measure the mRNA abundance of specific regions of the transcript, which were normalized to RPLP0 mRNA level. Data are presented in relation to the expression level of the same transcript in male rats. The obtained data were analyzed using Student's T-test and bars represent the mean ± S.E. Asterisks indicate statistically significant differences versus male (*) whereas circles (¤) indicate significant effects of GHi in old males (p < 0.05).

In attempts to find putative hormonal factors behind the female-predominant expression of CD36 in rat liver, the hormonal states described above were investigated regarding hepatic CD36 mRNA expression. Expression levels of exons 1a-3, 1b-3, and 3–4 were compared between "feminized" and untreated (young) adult males. As demonstrated in Figure 4A, all transcripts were significantly increased in "feminized" males, except for exon 1b-3 in 17α-ethinylestradiol (EE) treated males. The feminizing effect was highest for exon 1a-3, suggesting that that EE and GHc treatment might induce CD36 expression preferentially through the exon 1a promoter whereas the changes associated with old age might have another mechanism of action.

When old male rats were treated by episodic administration of GH (GHi), which mimics the endogenous secretory pattern of GH in young adult male rats, *Cd36 *gene expression was repressed and restored to the same level as in young adult males (Figure 4B). However, exon specific repression by GHi treatment was not observed. This is in line with the finding that old age induced exons 1a-3, 1b-3, and 3–4 to similar extent (Figure 4A and 4B). This suggests that the low level of *Cd36 *expression in (young adult) male rats might be due to the pulsatile secretion of GH in males. When this pattern of GH secretion becomes less pulsatile (regarding the pulse amplitude) as the male rats get older, this inhibition is released and CD36 mRNA levels increase. We speculate that regulatory DNA sequences mediating the inhibitory action of pulsatile GH are present in both exon 1a and 1b promoters, whereas those important for the stimulatory effect of continuous GH are specific for exon 1a.

Since our studies were performed on whole animals it is impossible to determine whether the hormonal effects on CD36 expression are due to direct effects on the liver or through secondary changes. The dose of EE used in this study has been shown to "feminize" the male rat liver [23]. The relatively weak effect of this treatment might be due to an indirect action on the liver, with estrogen acting on the pituitary by altering the pattern of GH secretion. Estrogen treatment of male rats is known to "feminize" the secretory pattern of GH into a more continuous mode [24]. However, the greater effect obtained by GHc treatment compared to estrogen, could also be explained by other indirect effects of GH treatment. GH is for instance well-known to stimulate adipose lipolysis, leading to increased levels of circulating free fatty acids [25], which might reprogram hepatic gene expression through various lipid-modulated transcription factors.

### Nutritional regulation of CD36 expression

**Figure 5:**
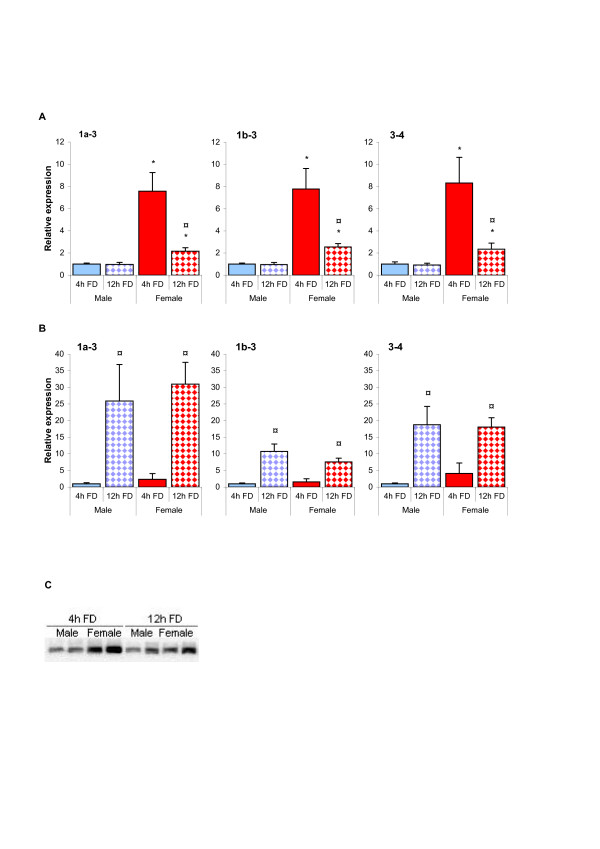
**Effects of food deprivation on CD36 expression in liver or skeletal muscle from male and female rats**. mRNA expression of hepatic (A) or skeletal muscle (B) CD36 was quantified by real-time PCR in male and female rats after 4 or 12 hours of food deprivation (FD). Exon-specific primers were used to measure the mRNA abundance of specific regions of the transcript, which were normalized to the mRNA level of RPLP0 in liver and that of ARBP in muscle. Data are presented in relation to the expression level of the same transcript in male rats after 4 hours of FD. The obtained data were analyzed using Student's T-test and bars represent the mean ± S.E. Asterisks (*) indicate significant sex differences whereas circles (¤) indicate significant effects of starvation (p < 0.05) Protein expression of hepatic CD36 in male and female rats after 4 or 12 hours of FD using immunoblot (C).

The nutritional status of the individual has been shown to affect CD36 expression in skeletal muscle, including starvation-mediated up-regulation [7]. Both starvation and increased secretion of GH will lead to activation of lipolysis in the adipose tissue, increased levels of circulating fatty acids, as well as increased expression of CD36 in muscle (starvation) or liver (GH). To determine whether the level of CD36 expression and thus ability to import LCFA is coordinated between muscle and liver, we compared these tissues regarding the different CD36 transcripts in animals deprived of food for either 4 or 12 hours. Animals that have been without food for 12 hours (fasting) should have an increased lipolysis in comparison to 4 hours. As demonstrated in Figure 5A, 12 h of food deprivation led to significantly reduced CD36 mRNA expression in livers from females, without any differences in alternative exon 1 transcripts. No effect was observed in male rats. As a consequence, the sex difference in CD36 expression was greatly reduced in the fasted rats. In contrast, the expression of CD36 in skeletal muscle was increased in both males and females (Figure 5B). This stimulatory effect of food withdrawal was highest for exon 1a-3, suggesting that fasting might induce CD36 expression preferentially through the exon 1a promoter. These observations confirms previous reports on *Cd36 *regulation in skeletal muscle [7], and shows that CD36 expression is differently regulated in muscle and liver. As demonstrated in Figure 5C, the starvation-mediated down-regulation of CD36 in female livers could also be observed at the protein level, indicating that animals in this nutritional state might prioritize muscular uptake of LCFA at the expense of hepatic.

### In silico promoter analysis of the rat Cd36 promoter

As described above, the gene structure of *Cd36 *in rat is similar to that described in mouse, with at least two alternative first exons (exon 1a and 1b). We observe a female-predominant expression of the transcripts starting with both exon 1a and exon 1b, but the sex-specific expression difference is greater for the transcripts starting with exon 1a. The exon 1a-specific up-regulation of CD36 is even more pronounced after GHc-induction in male rat liver. The effects of old age and starvation in liver, on the other hand, appeared to be independent of alternative first exon usage. We therefore wanted to identify putative transcription factor binding sites (TFBS) within the exon 1a and 1b promoters which might be able to explain the observed differences and similarities regarding first exon usage in rat liver.

**Figure 6:**
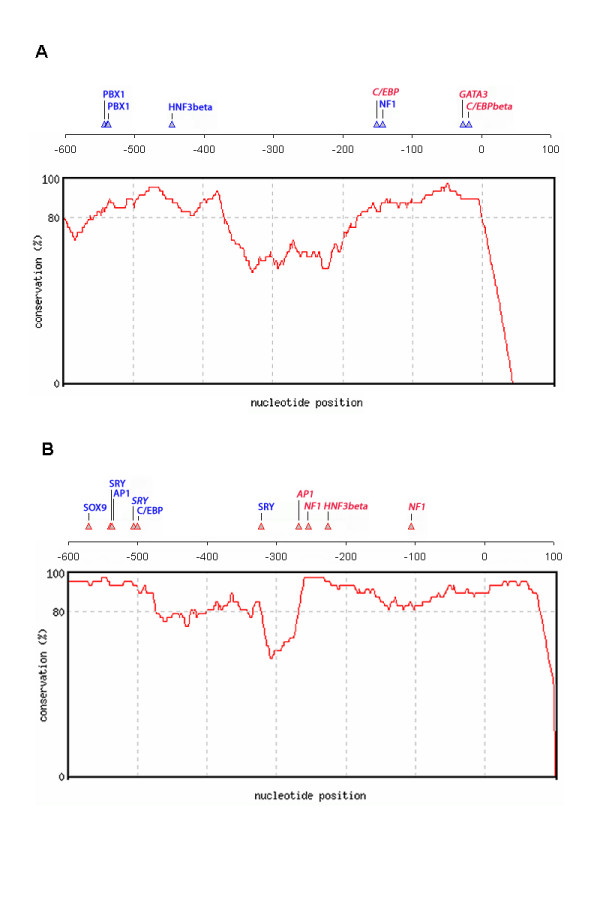
**Putative transcription factor binding sites within the alternative *Cd36 *1a and 1b promoters**. Selected liver- or sex-specific putative transcription factor binding sites (TFBS) were predicted in the most conserved regions (600 bp upstream to 50 bp downstream of TSS) of rat *Cd36 *1a (A) and 1b (B) promoters, using the Match™ program (available from the TRANSFAC^® ^website). All TFBS indicated in the figure have more than 90% similarity score in both core match and matrix match. The degree of sequence conservation between rat and mouse sequences within these two promoter regions is indicated in the figure. Putative TFBS in blue and bold are conserved between mouse and rat, whereas those in red, bold and italic are conserved among human, mouse and rat. See additional file for the exact positions of all putative TFBS, the corresponding scores, matrix identifiers and sequences.

We used the Match™ program, in which position specific weight matrix (PWM) models are used to predict TFBS, and the Consite program to identify evolutionary conserved sequences between rat, mouse and human in the upstream regions of the alternative first exons of CD36. The sequences from -600 to +50 base pairs relative to the transcription start sites of the two exons were found to be highly conserved between species (see additional file), so we focused the *in silico *analysis on these regions. PWM models of TFBS have been shown to predict sites that are functional *in vitro *[26] but it is also well known that the method results in a high number of predictions that are non-functional *in vivo *[27]. In order to make as relevant predictions as possible, we limited our analysis to available PWMs for transcription factors known to be expressed in the liver (GATA3, NF1 and PBX1) and transcription factors that are known from the literature to be sex-dependent or controlled by GH (AP-1, C/EBPβ, HNF3β, SOX9, SRY and STAT5, reviewed by Waxman and O'Connor 2006 [28]). Predicted binding sites for the available transcription factors are shown in Figures 6A (exon 1a) and 6B (exon 1b). Most of the putative TFBS were conserved between rat and mouse and some could also be predicted in the human promoters. Please refer to additional files for sequences, positions and other information related to these putative TFBS. Some TFBS were found to be common between the two promoters, such as those for HNF3β, whereas others were promoter-specific. The two putative binding sites for HNF3β were both found to be conserved between rat and mouse and might be of special interest since constitutively active HNF3β was previously shown to increase hepatic CD36 expression [13]. Furthermore, the level of HNF3β in rat liver has been shown to be GH-dependent [29] and to induce the expression of the female-specific *Cyp2c12 *transcript in rat liver [30]. Needless to say, this does not imply that CD36 expression is regulated in the same manner as *Cyp2c12*.

Since exon 1a containing transcripts were shown to increase more in response to GHc, there might be inhibitory factors operating within the exon 1b promoter. STAT5b is a candidate repressor as it is activated in male rat liver in direct response to each incoming plasma GH pulse and is partially down-regulated by a continuous, female-like pattern of GH-stimulation [31,32]. STAT5b has been proposed to collaborate with the HNFs to regulate the sex-dependent hepatic expression of *cyp*-genes [30,33]. No putative STAT5b binding sites were however revealed within either promoter. Binding sites for the male-specific transcription factors Sry (sex-determining region on the Y chromosome) and SOX9 (Sry-related HMG box-9) were identified within the exon 1b promoter. Overexpression of the SRY-like protein IRE-ABP (SOX4) has been shown to inhibit the stimulatory effect of C/EBPα on *Cyp2c12 *expression in primary hepatocytes [34]. Purely speculative, similar mechanism might operate within the exon 1b promoter, which could explain the differences in the amount of up-regulation of exon 1a and exon 1b in GHc treated male rats.

As mentioned above, PWMs have been shown to produce a lot of false positive binding site predictions. Potential sites must therefore be validated experimentally before any conclusions can be drawn about which transcription factors mediate the up- or down-regulation of the alternative first exons of CD36 observed in this study. Nevertheless, these predictions are based on the currently available tools for *in silico *predictions of regulatory elements, and can be considered a first guess and a guide to which sites to prioritize for further *in vitro *and *in vivo *studies.

## Conclusion

The aim of this study was to improve our knowledge about regulatory mechanisms behind hepatic *Cd36 *expression. We have extended the sequence information of the rat *Cd36 *5'-UTR and shown that the gene structure of *Cd36 *in rat is similar to that described in mouse, which contains at least two alternative first exons (exon 1a and 1b). The rat *Cd36 *exon 1a promoter was sequenced and shown to be highly similar to human and murine *Cd36*. A greater sex-specific expression difference was observed for the transcripts starting with exon 1a in rat liver. Furthermore, estrogen or GHc treatment of male rats induced CD36 expression preferentially through the exon 1a promoter. Old age was also associated with increased CD36 expression in male rats, albeit without any preferential first exon usage. GHi treatment in old male rats reversed (reduced) this effect. Similarly, the two different exon 1 containing transcripts were equally well reduced in response to starvation in female rats. The results obtained in this study confirm and extend our previous observation that GH is an important regulator of hepatic CD36, and depending on the mode of treatment (continuous or intermittent) the gene might be either induced or repressed. We therefore suggest that the effects of continuous GH secretion in females (which is stimulatory) and intermittent GH secretion in males (which is inhibitory) explains the sex-different expression of this gene.

## Methods

### Animals and treatments

Male (n = 5) and female (n = 6) Sprague-Dawley rats (ten weeks of age) and C57BL/6 mice (six of each sex) were maintained under standardized conditions. Food restricted animals, in groups of four, were either without chow from 11 PM to 11 AM (12 h food withdrawal), or from 7 AM to 11 AM (4 h food withdrawal). Where indicated, five male rats (ten weeks of age) were treated with bovine GH (a kind gift from Pharmacia and Upjohn AB) by continuous infusion from osmotic minipumps, as described in [16]. Old (18 months of age) male rats were injected twice daily with GH (n = 5) or vehicle (n = 4), as described in[21]. Six young animals (ten weeks of age) were injected subcutaneously once daily with 17α-ethinylestradiol (EE) (Sigma, E-4876), as described previously [23]. Control animals (n = 5) received equivalent amounts of the vehicle alone. After one week of treatments, animals were anesthetized and sacrificed. Tissues were removed, immediately frozen in liquid nitrogen, and stored at -80°C until further analysis. All animal experiments were approved by the institutional animal care and use committee.

### RNA isolation, cDNA synthesis and real-time PCR

Total RNA was extracted from frozen livers or skeletal muscle with TRIzol^®^Reagent (Invitrogen Life Technologies). Before reverse transcription, DNA was removed by RQ1 RNase-free DNAse (Promega). From 5 μg of total RNA, the first strand cDNA was made using iScript™ cDNA synthesis kit (Bio-Rad Laboratories, CA). Exon-specific primers were designed by the Primer3 program [35] and synthesized by Thermo, as summarized in Table 1. The setup of primers is presented in Figure 2B. The following reference genes were used; ribosomal protein large P0 (*Rplp0*) for rat liver; acidic ribosomal phosphoprotein P0 (*Arbp*) for rat muscle; glyceraldehyde-3-phosphate dehydrogenase (*Gapdh*) for mouse liver.

**Table 1:** Primer sequences for real-time PCR analysis of CD36 mRNA expression

	**Rat**	**Mouse**
exon 1a forward	TGCAACTGCATTTTGATGGT	
exon 1b forward	GGAGCAACTGGTGAATGGTT	
exon 3 reverse	TGCTTCTTGCCAACTCACAG	
exon 3 forward	AACCAGGCCACATAGAAAGC	TGTGTTTGGAGGCATTCTCA
exon 4 reverse	AAGGACCTCTCTGTTTAACCTTGA	TTTTGCACGTCAAAGATCCA

The *Cd36 *exon 1a forward primer was designed from rat EST [GenBank:AB005743] and mouse EST [GenBank:BC010262]. Primers for *Cd36 *exon 1b forward and exon 3 reverse were designed from rat [GenBank:AW919183] and mouse [GenBank:NM_007643]. [GenBank:NM_007643] was also used to design *Cd36 *exon 3 forward and exon 4 reverse in mouse.

Real-time PCR was performed from first strand cDNA with iQ™ SYBR^® ^Green Supermix (Bio-Rad Laboratories, CA) in DNA Engine Opticon™2 System (MJ Research, CA). Relative standard curves were constructed using serial dilutions of pooled cDNA representing all individual hepatic RNA samples used in this study. The specific transcript levels were determined using Opticon Monitor 3, normalized with the level of the corresponding reference gene in each sample, and the obtained data were analyzed using Student's T-test. We observed a certain variation in the absolute mRNA levels. At the present we suspect that the nutritional state of the individual animals might contribute to this.

### Sequencing and sequence alignment

Primers used to clone the promoter of exon 1a are left primers in the genomic region, 5'-GAGCAGTTCATTTTTAAGCTG-3' and 5'-AAGGGGATTGCTGTGA-3', and right primer in exon 1a 5'-ACCATCAAAATGCAGTTG-3'. For exon 1a, 1b to exon 3, primers used are the same as for the real-time PCR. Exon 1a, 1b and the promoter of exon 1a were amplified by PCR using standard conditions and sequenced in duplicates using BigDye™ reagents (Amersham Biosciences) on an ABI sequencer. Multiple sequence alignment was then performed by the program Clustal W version 1.83 [36].

### In silico promoter analysis

Orthologous promoter sequences from human, mouse and rat *Cd36 *were extracted from the UCSC genome browser and [GenBank:AF317787]. Pairwise alignment with the ORCA algorithm was used to identify conserved regions by CONSITE [37]. Putative transcription factor binding sites (TFBS) were predicted in the sequences for promoter 1a and 1b by Match™ program public version 1.0 [38]. The program uses a library of mononucleotide weight matrices from TRANSFAC^® ^6.0. We limited our analysis to matrices corresponding to transcription factors known to be expressed in liver (GATA3, NF1 and PBX1) or known to be sex- or GH dependent (AP-1, C/EBPβ, HNF3β, SOX9, SRY and STAT5). The PWM for STAT5 was found in a publication by Ehret et al. [39] and analyzed separately. We reported TFBS predictions that gave a relative score of at least 90% of the best possible match between the matrix model and the DNA sequence. See additional files for more information.

### Immunoblotting

Whole liver cell lysates were obtained by homogenizing 100 mg of liver in 1 ml RIPA buffer (50 mM Tris-HCl, pH 7.4, 1% Triton X-100, 150 mM NaCl, 5 mM EDTA, 1 mM PMSF, 1 mM Na_3_VO_4_, 10 mM NaF, 1 μg/ml of aprotinin, leupeptin, and pepstatin), using a polytrone PT-2000 (Kinematica AG), followed by 20 minutes of centrifugation (12 000 g). The resulting supernatants were collected and proteins resolved by SDS-PAGE on PVDF membrane. The membranes were blocked for 1 h in Tris-buffered saline (TBS; 10 mM Tris pH 8.0, 150 mM NaCl) containing 0.1% (v/v) Tween-20 and 5% (w/v) milkpowder (TBS-MILK), incubated overnight with monoclonal anti-CD36 antibodies (anti murine CD36, Cascade Biosciences) diluted 1:2500 in TBS-MILK, washed and incubated with the secondary antibody (1:10000) (goat anti-mouse IgG, Santa Cruz, CA). After additional washing steps antibody binding was visualized using an ECL detection system (Pierce Biotechnology, Inc).

## Authors' contributions

LC took part in the design of the study, carried out animal experiments, RNA isolation, cDNA synthesis and real-time PCR analyses, sequencing and sequence alignments, in silico promoter analysis, immunoblotting and drafting of the manuscript. MA and JO took part in the design of the study and bioinformatics analyses. CG took part in animal experiments, carried out RNA isolation, cDNA synthesis and real-time PCR analyses. LFP carried out animal experiments. GN participated in design and coordination of the study, took part in animal experiments and helped to draft the manuscript. PTE participated in design and coordination of the study, took part in animal experiments and drafted the manuscript. All authors read and approved the final manuscript.

## Supplementary Material

Additional file 1 *in silico *promoter analysis of *Cd36 *exon 1a promoter and exon 1b promoter. The data provided show the details of the putative TFBS on the studied DNA sequences. Click here for file
