# Supplementary material for: Hormonal and nutritional regulation of alternative CD36 transcripts in rat liver – a role for growth hormone in alternative exon usage
Source: BMC Mol Biol. 2007 Jul 17;8:60. doi: 10.1186/1471-2199-8-60 (PMC1934915; doi:10.1186/1471-2199-8-60)
Supplement: Additional file 1 — in silico promoter analysis of Cd36 exon 1a promoter and exon 1b promoter. The data provided show the details of the putative TFBS on the studied DNA sequences. [file 1471-2199-8-60-S1.doc]

**Additional File**

***in silico* promoter analysis of *Cd36* exon 1a promoter and exon 1b promoter**

Orthologous promoter sequences from human, mouse and rat *Cd36* were extracted from the UCSC genome browser and the rat promoter [GenBank:AF317787]. Promoters of exon 1a for mouse and human were defined as 5000bp upstream and 100bp downstream of transcription start sites (TSS) in human [GenBank:CB958830] and mouse [GenBank:BC010262] in the UCSC genome browser. Since the genome information in rat exon 1a and its promoter was missing in genome databases, the rat *Cd36* promoter [GenBank:AF317787] (2246bp) was used for the analysis. TSS is defined as the fourth base in [GenBank:AB005743], due to inconsistence between the first three bases and our sequence data. Promoters of exon 1b in human, mouse and rat, 5000bp upstream and 100bp downstream of TSS in [GenBank:NM_00100548], [GenBank:NM_007643] and [GenBank:AW919183] respectively, were extracted in a similar manner.

Conserved regions were identified by CONSITE using pairwise alignment with the ORCA algorithm (http://mordor.cgb.ki.se/cgi-bin/CONSITE/consite). The most conserved region between human, mouse and rat was then identified as 600bp upstream and 50bp downstream of TSS. Figure 1-4 illustrate the sequence conservation between human and mouse or between mouse and rat in these promoter regions. TSS is presented as position 5001 in the figures.

Putative transcription factor binding sites (TFBS) were predicted by Match^TM^ program public version 1.0 (http://www.gene-regulation.com/cgi-bin/pub/programs/match/bin/match.cgi?). The program uses a library of mononucleotide weight matrices from TRANSFAC® 6.0. We

limited our analysis to matrices corresponding to transcription factors known to be expressed in liver (GATA3, NF1 and PBX1) or known to be sex- or GH dependent (AP-1, C/EBP_, HNF3_, SOX9, SRY and STAT5). We reported TFBS predictions that gave a relative score of at least 90% of the best possible match between the matrix model and the DNA sequence. Information for the putative TFBS in mouse and human is also shown where the sites were conserved (shaded). Table 1 and 2 show the detailed information of these putative TFBS.

**Table 1. Detailed information of putative TFBS in *Cd36* exon 1a promoter**

| **Transcription factor** | **Position** | **Strand** | **Core Match** | **Matrix match** | **Sequence** | **Matrix Identifier** | **Species** |
| --- | --- | --- | --- | --- | --- | --- | --- |
| Pbx-1 | -544 | (+) | 1 | 0.96 | aagAATCAa | V$PBX1_01 | Rat |
| Pbx-1 | -494 | (+) | 1 | 0.96 | aagAATCAa | V$PBX1_01 | Mouse |
| Pbx-1 | -540 | (+) | 0.948 | 0.961 | atcAATAAa | V$PBX1_01 | Rat |
| Pbx-1 | -490 | (+) | 0.948 | 0.961 | atcAATAAa | V$PBX1_01 | Mouse |
| HNF-3 beta | -447 | (+) | 1 | 0.933 | tggaaTGTTTcccta | V$HNF3B_01 | Rat |
| HNF-3 beta | -396 | (+) | 1 | 0.924 | tggagTGTTTcccta | V$HNF3B_01 | Mouse |
| C/EBP | -151 | (-) | 0.965 | 0.956 | aggttaACAAAag | V$CEBP_01 | Rat |
| C/EBP | -168 | (-) | 0.972 | 0.967 | aggttaGCAAAtg | V$CEBP_01 | Mouse |
| C/EBP | -145 | (-) | 0.965 | 0.971 | aggttaACAAAca | V$CEBP_01 | Human |
| NF-1 | -143 | (-) | 0.921 | 0.906 | aaaagctggatGCCAGgc | V$NF1_Q6 | Rat |
| NF-1 | -160 | (-) | 0.921 | 0.905 | aaatgctggatGCCAGgt | V$NF1_Q6 | Mouse |
| GATA-3 | -27 | (+) | 0.968 | 0.909 | agtGATTTga | V$GATA3_03 | Rat |
| GATA-3 | -44 | (+) | 0.968 | 0.909 | agtGATTTga | V$GATA3_03 | Mouse |
| GATA-3 | -21 | (+) | 0.968 | 0.909 | agtGATTTga | V$GATA3_03 | Human |
| C/EBP beta | -18 | (-) | 0.994 | 0.919 | acttggGGCAAaaa | V$CEBPB_02 | Rat |
| C/EBP beta | -35 | (-) | 0.994 | 0.902 | acttgtGGCAAaca | V$CEBPB_02 | Mouse |
| C/EBP beta | -5 | (-) | 0.994 | 0.917 | acttggGGCAAaca | V$CEBPB_02 | Human |

**Table 2. Detailed information of putative TFBS in *Cd36* exon 1b promoter**

| **Transcription factor** | **Position** | **Strand** | **Core Match** | **Matrix match** | **Sequence** | **Matrix Identifier** | **Species** |
| --- | --- | --- | --- | --- | --- | --- | --- |
| SOX-9 | -571 | (+) | 1 | 1 | ttagaACAATgggg | V$SOX9_B1 | Rat |
| SOX-9 | -574 | (+) | 1 | 0.999 | ctagaACAATgggc | V$SOX9_B1 | Mouse |
| SRY | -539 | (-) | 1 | 1 | ttTGTTT | V$SRY_01 | Rat |
| SRY | -543 | (-) | 1 | 1 | ttTGTTT | V$SRY_01 | Mouse |
| AP-1 | -537 | (-) | 0.935 | 0.919 | tgttTGTCAtc | V$AP1_Q4 | Rat |
| AP-1 | -541 | (-) | 0.935 | 0.919 | tgttTGTCAtc | V$AP1_Q4 | Mouse |
| SRY | -506 | (-) | 1 | 0.983 | ccaATTGTtttc | V$SRY_02 | Rat |
| SRY | -509 | (-) | 1 | 0.983 | ccaATTGTtttc | V$SRY_02 | Mouse |
| SRY | -543 | (-) | 1 | 1 | ttTGTTT | V$SRY_01 | Mouse |
| C/EBP | -501 | (-) | 1 | 0.977 | tgttttCCAAAtt | V$CEBP_01 | Rat |
| C/EBP | -504 | (-) | 1 | 0.977 | tgttttCCAAAtt | V$CEBP_01 | Mouse |
| SRY | -322 | (+) | 1 | 1 | AAACAaa | V$SRY_01 | Rat |
| SRY | -324 | (+) | 1 | 1 | AAACAaa | V$SRY_01 | Mouse |
| AP-1 | -268 | (+) | 1 | 0.952 | tcTGACTtact | V$AP1_Q4 | Rat |
| AP-1 | -257 | (+) | 1 | 0.952 | tcTGACTtact | V$AP1_Q4 | Mouse |
| AP-1 | -266 | (+) | 1 | 0.952 | tcTGACTtact | V$AP1_Q4 | Human |
| NF-1 | -254 | (-) | 1 | 0.984 | atgggaacataGCCAAaa | V$NF1_Q6 | Rat |
| NF-1 | -243 | (-) | 1 | 0.982 | atgggaacatgGCCAAaa | V$NF1_Q6 | Mouse |
| NF-1 | -253 | (-) | 1 | 0.984 | gatgggaaataGCCAAaa | V$NF1_Q6 | Human |
| HNF-3 beta | -226 | (-) | 0.93 | 0.905 | aaaaaAAAGActgct | V$HNF3B_01 | Rat |
| HNF-3 beta | -221 | (-) | 0.93 | 0.905 | aaaaaAAAGActgct | V$HNF3B_01 | Mouse |
| HNF-3 beta | -236 | (-) | 0.93 | 0.91 | aaaaaAAAAAatgct | V$HNF3B_01 | Human |
| NF-1 | -106 | (+) | 1 | 0.992 | tgTTGGCaacaaaccaca | V$NF1_Q6 | Rat |
| NF-1 | -102 | (+) | 1 | 0.992 | ccTTGGCaaccaaccaca | V$NF1_Q6 | Mouse |
| NF-1 | -114 | (+) | 0.921 | 0.918 | ctCTGGCaacaaaccaca | V$NF1_Q6 | Human |

**A**

**
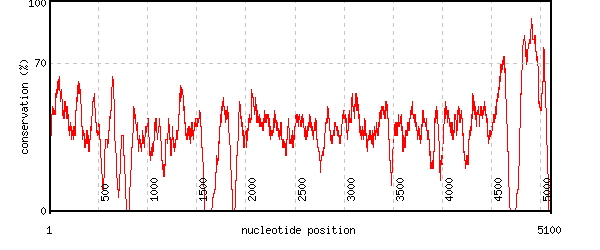
**

**B**


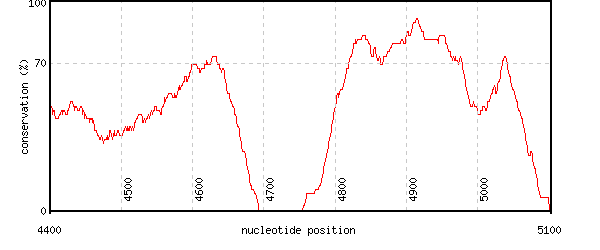


**Figure 1.** Sequence conservation of promoter 1a in human when compared with that of mouse.

A. The entire sequence, -5000bp to +100bp of TSS. B. The most conserved region, -600bp to 50bp of TSS. TSS is presented as position 5001.

**A**

**
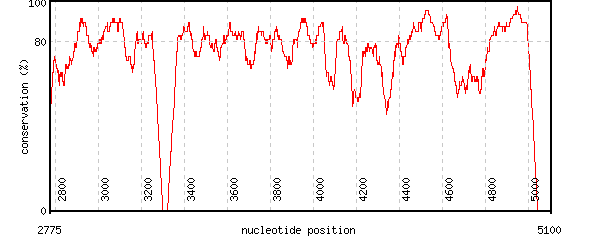
**

**B**


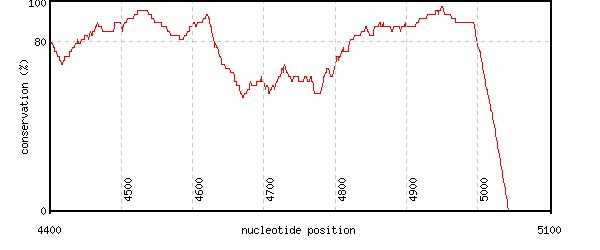


**Figure 2.** Sequence conservation of promoter 1a in rat when compared with that of mouse.

A. The entire sequence, -2225bp to +100bp of TSS. B. The most conserved region, -600bp to 50bp of TSS. TSS is presented as position 5001.

**A**

**
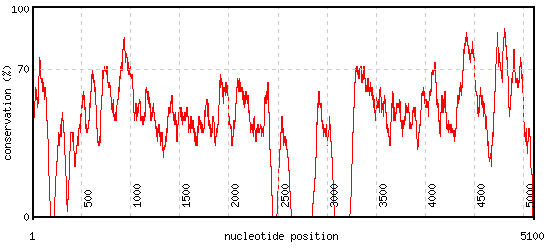
**

**B**


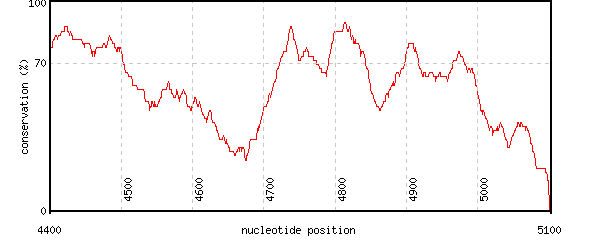


**Figure 3.** Sequence conservation of promoter 1b in human when compared with that of mouse.

A. The entire sequence, -5000bp to +100bp of TSS. B. The most conserved region, -600bp to 50bp of TSS. TSS is presented as position 5001.

**A**

**
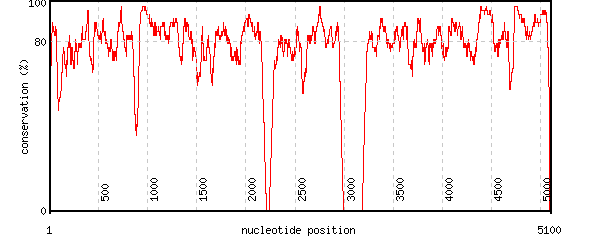
**

**B**


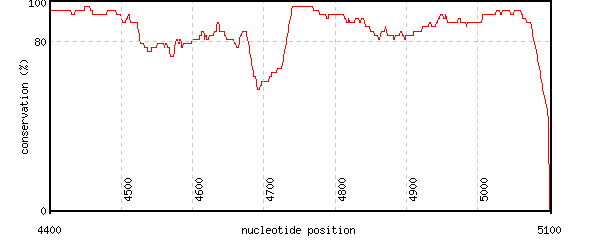


**Figure 4.** Sequence conservation of promoter 1b in rat when compared with that of mouse.

A. The entire sequence, -5000bp to +100bp of TSS. B. The most conserved region, -600bp to 50bp of TSS. TSS is presented as position 5001.
